# Regulation of the adaptation to ER stress by KLF4 facilitates melanoma cell metastasis via upregulating NUCB2 expression

**DOI:** 10.1186/s13046-018-0842-z

**Published:** 2018-07-28

**Authors:** Dongmei Zhang, Jingrong Lin, Yulin Chao, Lu Zhang, Lei Jin, Na Li, Ruiping He, Binbin Ma, Wenzhi Zhao, Chuanchun Han

**Affiliations:** 1 Institute of Cancer Stem Cell, Dalian Medical University, Dalian, 116044 China; 2 Department of Orthopedics, Second Affiliated Hospital, Dalian Medical University, Dalian, 116044 China; 3 Department of Physiology, College of Basic Medical Sciences, Dalian Medical University, Dalian, 116044 China; 4 Department of Dermatology, the First Affiliated Hospital, Dalian Medical University, Liaoning, 116027 China

**Keywords:** Melanoma, ER stress, Apoptosis, KLF4, Metastasis

## Abstract

**Background:**

Adaptation to ER stress has been indicated to play an important role in resistance to therapy in human melanoma. However, the relationship between adaptation to ER stress and cell metastasis in human melanoma remains unclear.

**Methods:**

The relationship of adaptation to ER stress and cell metastasis was investigated using transwell and mouse metastasis assays. The potential molecular mechanism of KLF4 in regulating the adaptation to ER stress and cell metastasis was investigated using RNA sequencing analysis, q-RT-PCR and western blot assays. The transcriptional regulation of nucleobindin 2 (NUCB2) by KLF4 was identified using bioinformatic analysis, luciferase assay, and chromatin immunoprecipitation (ChIP). The clinical significance of KLF4 and NUCB2 was based on human tissue microarray (TMA) analysis.

**Results:**

Here, we demonstrated that KLF4 was induced by ER stress in melanoma cells, and increased KLF4 inhibited cell apoptosis and promoted cell metastasis. Further mechanistic studies revealed that KLF4 directly bound to the promoter of NUCB2, facilitating its transcription. Additionally, an increase in KLF4 promoted melanoma ER stress resistance, tumour growth and cell metastasis by regulating NCUB2 expression in vitro and in vivo. Elevated KLF4 was found in human melanoma tissues, which was associated with NUCB2 expression.

**Conclusion:**

Our data revealed that the promotion of ER stress resistance via the KLF4-NUCB2 axis is essential for melanoma cell metastasis, and KLF4 may be a promising specific target for melanoma therapy.

**Electronic supplementary material:**

The online version of this article (10.1186/s13046-018-0842-z) contains supplementary material, which is available to authorized users.

## Background

Melanoma is the most aggressive skin cancer and is associated with a high mortality rate [1]. In the past, multiple strategies have been used for melanoma treatment. For primary melanoma, surgical resection was the best option and the patients have a good post-treatment prognosis. For metastatic melanoma, only a few options were clinically available for treating the disease such as targeting BRAF and MEK using small molecule inhibitors, immunotherapeutic antibodies against the immune checkpoints T-lymphocyte-associated antigen 4 (CTLA-4) and programmed cell-death protein 1 (PD-1) and the modified oncolytic herpes virus talimogene laharparepvec (T-VEC) and the 5-year survival rate of patients has been improved to some extent [2–14]. However, similar to other cancers, distant metastasis is still a major obstacle to the treatment of melanoma. Thus, understanding the potential molecular mechanisms of tumour metastasis is important for improving the overall prognosis of patients with melanoma.

Metastasis is a multi-step process releasing tumour cells from a primary lesion to a disparate organ or organs within the body. Tumour cells change their characteristics throughout the process, which enables them to proliferate and migrate, invading surrounding tissues [15]. When tumours metastasise, the cells undergone diverse microenvironments such as hypoxia, nutrient starvation, leading to endoplasmic reticulum (ER) stress [16].

Previous studies have indicated that melanoma largely adapts to ER stress depending on a series of potential molecular mechanisms including upregulation of the antiapoptotic Bcl-2 family protein Mcl-1 by miR-149* or activation of autophagy by RIPK1 [17–19]. Our recent study indicated that the increase of cancer stem cells by HOXB9 inhibited ER stress-induced apoptosis in melanoma cells [20]. Although the adaptation to ER stress has been reported to play a role in the resistance of melanoma cells to various therapeutic agents, the role of adaptation to ER stress in melanoma metastasis is still unclear.

Here, we found that KLF4, a zinc finger-type transcription factor, was induced by ER stress, leading to the inhibition of cell apoptosis and the promotion of cell metastasis in melanoma. Further mechanistic studies revealed that KLF4 directly bound to the promoter of NUCB2 and facilitated its transcription. In addition, KLF4 promoted melanoma ER stress resistance, tumour growth and cell metastasis by regulating NCUB2 expression in vitro and in vivo. Elevated KLF4 was found in human melanoma tissues, which was associated with NUCB2 upregulation. Therefore, our finding suggests that the KLF4-NUCB2 pathway may be an important apparatus in melanoma metastasis under ER stress.

## Methods

### Cell culture and reagents

Human melanoma cell lines Me1007, Mel-RM, A375, Mel-CV, and SK-Mel-28 were obtained as previously described [21]. Studies on a panel of melanoma cell lines revealed that ER stress did not induced significantly apoptosis in Mel-RM, A375, Mel-CV, SK-Mel-28 but Me1007. Me1007 was reported to be a ER stress non-resistant melanoma cell line [22]. These cells were cultured in DMEM containing 10% FBS (ExCell Bio, Lot: FSP500) and 0.1% Savelt™ (Hanbio Co. LTD., 1:1000). The following antibodies were used in this study: antibodies against GAPDH (Santa Cruz Biotechnology, Dallas, TX, USA; SC-25778, 1:1000); PARP (Santa Cruz Biotechnology, SC-8007, 1:1000); KLF4 (Santa Cruz Biotechnology, SC-20691, 1:10 for ChIP); KLF4 (Cell Signaling Technology, #12173S, 1:500); NUCB2 (Abcam, ab229683, 1:300); KLF8 (Abcam, ab168527, 1:300); KLF5 (Proteintech, 21,017–1-AP 1:500); KLF6 (Proteintech, 14,716–1-AP 1:500); GRP78 (Santa Cruz Biotechnology, SC-13968,1:1000). Tunicamycin (TM, Lot:T7765) and thapsigargin (TG, Lot:T9033) were purchased from Sigma Chemical Co. They was dissolved in DMSO and made up in stock solutions of 3 mmol/L for tunicamycin and 1 mmol/L for thapsigargin. The cells were treated with 3 μmol/L TM or 1 μmol/L TG as the indicated times.

### Plasmids and transfection

KLF4 and NUCB2 were constructed into pCDNA3.0-Flag vector. The lentiviral expression vector for KLF4 was constructed by inserting full-length cDNA into a pCDH vector. 5 μg plasmids were transfected into melanoma cells(1 × 10^6^) using Lipofectamine 3000 (Invitrogen, Carlsbad, CA, USA) according to the Lipofectamine™ 3000 Reagent Protocol; The empty vectors were used as the negative control. To generate lentivirus expressing KLF4, HEK 293 T cells grown on a 6 cm dish were transfected with 2 μg pCDH-KLF4 or control vector (pCDH), 1.5 μg psPax2, and 0.5 μg pMD2G. Twenty-four hour after the transfection, cells were cultured with DMEM containing 10% FBS for an additional 24 h. The culture medium containing lentiviral particles was centrifuged at 1000 g for 5 min. Viral particles collected in the supernatant were used for infection. In order to establish the stable cell line, the puromycin was used as a selection marker for the infected cells. The expression efficiency was evaluated by western blot analysis.

### RNA interference and KLF4 knockout cell

RNA interference was performed as previously described [20, 23]. The sequences targeting NUCB2–1 were: 5- CCACAGATTTAGATATGCTAA-3 and NUCB2–2 5-GCGTGAATATCATCAGGTCAT-3. KLF4–1, 5-ATCGGTCATCAGCGTCAGCAA-3. KLF4–2 5-AAGTCATCTTGTGAGTGGATAA-3.

KLF4 knockout was achieved by CRISPR/Cas9. sgRNA design and cloning were performed according to the Feng Zhang lab general cloning protocols. KLF4 sgRNAs oligos were designed based on the target site sequence (20 bp) and were flanked on the 3′ end by a 3 bp NGG PAM sequence. Using the Cas9 target design tools (http://www.genome-engineering.org), we designed two sgRNAs for each target: KLF4 sg1 UP:5-CACCCGCCGGGCCAGACGCGAACG-3. DN:5-AAACCGTTCGCGTCTGGCCCGGCG-3; Sg2 UP:5-CACCGTCTTTCTCCACGTTCGCGTC-3 DN:5-AAACGACGCGAACGTGGAGAAAGAC-3. The sgRNAs were cloned into the lentiCRISPRv2 vector (Addgene). For lentivirus production, cloned lentiCRISPRv2 plasmids were co-transfected into HEK293T cells with the packaging plasmids pVSVg (AddGene 8454) and psPAX2 (AddGene 12,260). The lentivirus was harvested, and Mel-RM cells were infected with two sgRNA mixtures for KLF4. Forty-eight hours after infection, the cells were placed under puromycin selection for 2 weeks, and the single-cell-derived clones were picked, expanded and knockout of KLF4 was verified by western blot analysis.

### Tissue microarrays and immunohistochemistry

Melanoma tissue microarrays were purchased from Alenabio (Xi’an, China), and they contained a total of 30 melanoma tissues and normal tissues. The immunohistochemistry was performed as previously described [24]. The characteristics of the patients and their tumours were collected through the review of medical records and pathologic reports. Informed consent with approval of the ethics committee of Taizhou Hospital of Zhejiang Province was obtained. All of the methods in this study were in accordance with the approved guidelines, and all of the experimental protocols were approved by the ethics committee of Taizhou Hospital of Zhejiang Province.

Sections were stained with Masson’s trichrome and H&E (haematoxylin and eosin) for histopathological examination. For immunohistochemistry, sections were subjected to antigen retrieval using microwave heating at 95 °C in citrate buffer (pH = 6.0, for KLF4 and NUCB2). The indicated antibodies specific for KLF4 (1:200) and NUCB2 (1:100) were diluted according to the manufacturer’s instructions. The degrees of immunostaining were reviewed and scored by two independent observers. The proportion of the stained cells and the extent of the staining were used as criteria of evaluation. For each case, at least 1000 tumor cells were analyzed. For each sample, the proportion of KLF4 and NUCB2-expressing cells varied from 0 to 100%, and the intensity of staining varied from weak to strong. One score was given according to the percentage of positive cells as:< 5% of the cells:1 point; 6–35% of the cells:2 point; 36–70% of the cells:3 point; > 70% of the cells: 4 point. Another score was given according to the intensity of staining as: negative staining: 1 point; weak staining (light yellow): 2 point; moderate staining (yellowish brown): 3 point; and strong staining (brown): 4 point. A final score was then calculated by multiple the above two scores. If the final score was equal or bigger than four, the protein expression in the tumor was considered high; otherwise, the protein expression in the tumor was considered low. The clinical information of patients with melanoma was provided in the Additional file 1.

### ChIP assay

The ChIP assay was performed as previously described [25].

Quantitative real-time polymerase chain reaction assay (q-RT-PCR) Total RNA was isolated using Trizol (Invitrogen). One microgram of total RNA was used to synthesize cDNA using the PrimeScript™ RT reagent kit (Takara, RR047A) according to the manufacturer’s instructions. The primers were as follows: NUCB2 up: 5- CCTGTGGAAAGTGCGAAGATAG -3, dn: 5- GCCTCCCACTCTTTATTTCCTC -3; and ACTIN up: 5-GACCTGACTGACTACCTCATGAAGAT-3, dn: 5-GTCACACTTCATGATGGAGTTGAAGG-3. KLF4 up: 5- ACCTACACAAAGAGTTCCCATC-3; dn: 5-TGTGTTTACGGTAGTGCCTG-3. KLF5 up: 5- GAAGGAGTAACCCCGATTTGG-3; dn:5- CTTCCCAGGTACACTTGTATGG-3; KLF6 up:5- CTTTAACGGCTGCAGGAAAG-3, dn 5- GGAAGTGCCTGGTTAACTCATC-3; KLF8 up: 5- CTCACCGCAGAATCCATACAG-3, dn 5- GCACCGAAAAGGCTTGATG-3.

### Promoter reporters and dual-luciferase assay

The promoter of NUCB2 and the matching mutant were constructed into the pGL3-basic vector. The luciferase activity was measured in a 1.5-ml Eppendorf tube with a Promega Dual-Luciferases Reporter Assay kit (Promega E1980) according to the manufacturer’s protocols after transfection. Relative Renilla luciferase activity was normalized to firefly luciferase activity. The assay was performed as previously described [26, 27].

### Cell migration assay

The cell migration assay was performed in a 24-well transwell plate with 8-mm polyethylene terephthalate membrane filters (Corning) separating the lower and upper culture chambers. In brief, melanoma cells were treated as indicated and plated in the upper chamber at 1 × 10^3^ cells per well in serum-free DMEM medium. The bottom chamber contained DMEM with 10% FBS. Cells were allowed to migrate for 24 h in a humidified chamber at 37 °C with 5% CO2. After the incubation period, the filter was removed and non-migrant cells on the upper side of the filter were detached using a cotton swab. Filters were fixed with 4% formaldehyde for 15 min, and cells located in the lower filter were stained with 0.1% crystal violet for 20 min and photographed.

Cells were counted in four randomly selected fields. Cell counts are expressed as the average number of cells per field of view. Three independent experiments were performed.

### Tumour growth and metastasis assay

Animal studies were conducted in accordance with the National Institute of Health Guide for the Care and Use of Laboratory Animals with the approval of the Animal Research Committee of Dalian Medical University. Male nude mice (4–6 weeks of age, 18–20 g) were obtained from the SPF Laboratory Animal Center of Dalian Medical University (Dalian, China) and were randomly divided into indicated groups. The mice (*n* = 6) in the groups were subcutaneously injected with the indicated cells or the injection was administered through the lateral tail vein. After 12 days, the tumour size was measured every 3 days by Vernier callipers and converted to TV according to the following formula: TV (mm^3^) = (axb^2^)/2, where a and b are the largest and smallest diameters, respectively. All animals were killed 4 weeks after injection, and the transplanted tumours were removed, weighed and fixed for further study. The weight of the lung and metastatic nodules were measured in the mice that were injected through the lateral tail vein. Lung was fixed with 4% formalin, and embedded in paraffin blocks. Sections of lung were stained with hematoxylin and eosin and photographed. The metastatic nodules were counted.

### Caspase-3 activity assay and cell viability assay

Caspase-3 activity was analysed using the Caspase-3 Activity Assay Kit (Roche) according to the manufacturer’s instructions. Briefly, the cell or tissue lysate was incubated with 100 μL of caspase-3 reagent at 37 °C for 1–2 h, and then the fluorescence intensity (at 370–425 nm excitation and 490–530 nm emission wavelengths) was measured using a Spectra MAX M5 spectrophotometer (Molecular Devices).

The cells were plated in 96-well plates at a density of 1000 cells in 200 μl of medium per well 24 h before the experiment. The cells were treated with TM as indicated in the figures, and the cell viability was determined using the CCK-8 kit (Cell Counting Kit-8).

### Statistics and data analyses

The data are expressed as the mean ± SD, and the statistical evaluation was performed using one-way analysis of variance (ANOVA). Values of *p* < 0.05 were considered statistically significant.

## Results

### ER stress-resistant melanoma cells possess a high metastatic ability

**Fig. 1:**
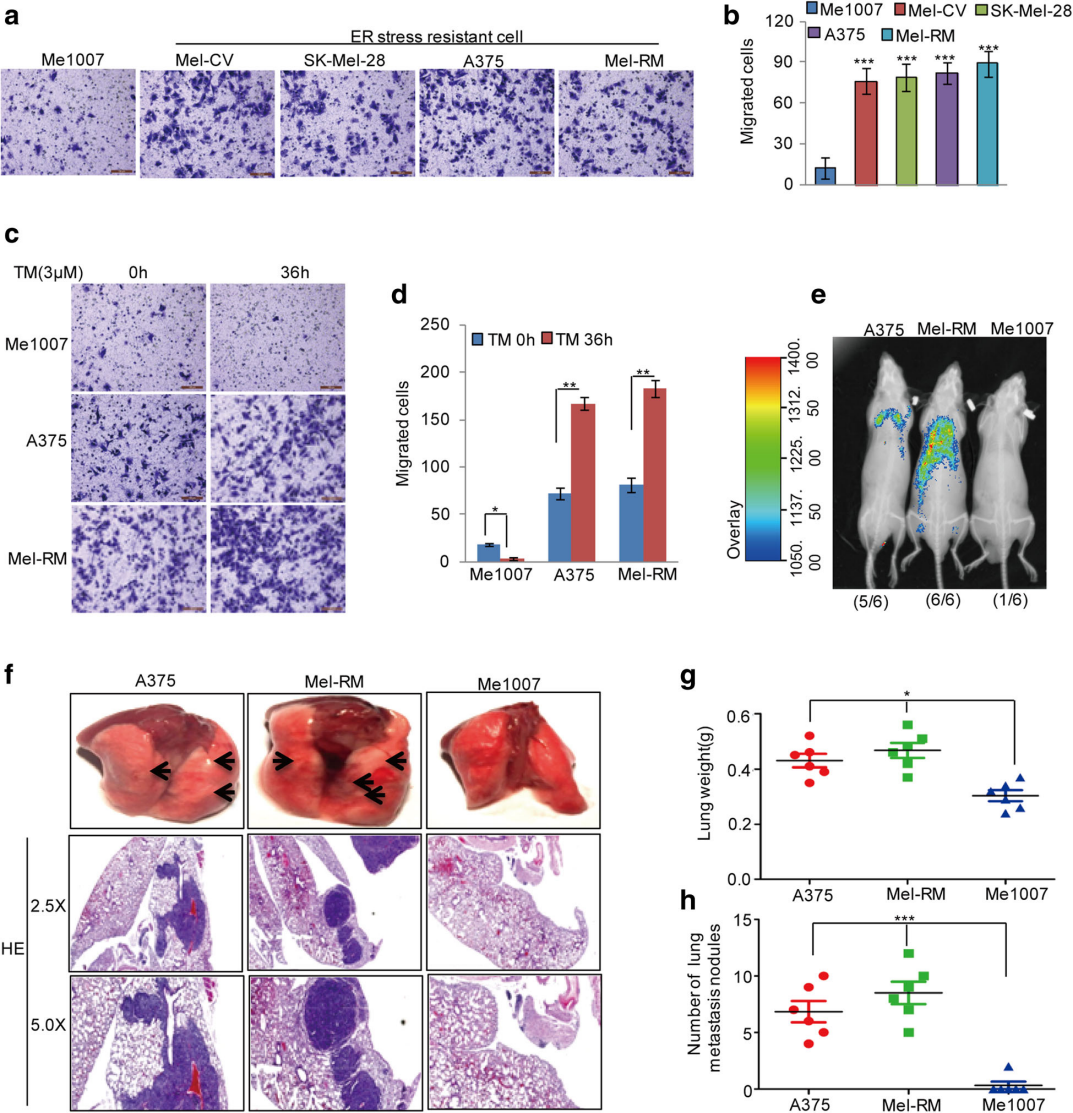
ER stress-resistant cells exhibited more migratory phenotype. **a-b** The migration abilities of the melanoma cells were detected by a transwell assay. The data represent the means ± SD of three independent experiments; ***p < 0.001 vs. control. **c-d** Me1007, A375 and Mel-RM cells were treated with 3 μM TM as indicated. Cell migration was analysed by a transwell assay. The data represent the means ± SD of three independent experiments; *p < 0.05,**p < 0.01 vs. control. **e** GFP-labelled Mel-RM, A375 and Me1007 cells (5 × 10^4^) were injected intravenously into nude mice (*n* = 6 in one group). After 4 weeks, the mice were subjected to bioluminescent imaging. **f-h** Representative images of the lung and HE pictures are shown (**f**), the weight and metastasis nodule in each group were calculated (**g-h**); *p < 0.05, ***p < 0.001 vs. control

To investigate the relationship between adaptation to ER stress and cell metastasis, we first compared the migratory capabilities of ER stress-sensitive melanoma cell Me1007 and ER stress-resistant melanoma cells Mel-CV, SK-Mel-28, A375 and Mel-RM. The ER stress-resistance phenotype of these melanoma cells was confirmed by the results of the CCK-8 assay (Additional file 2: Figure S1). We found that ER stress-resistant cells possessed a higher migratory capability than the ER stress-sensitive melanoma cell Me1007 (Fig. 1a and b). Subsequently, Me1007, Mel-RM, and A375 cells were treated with 3 μM tunicamycin (TM), which induced ER stress via the inhibition of glycosylation. The migratory abilities were examined using a transwell assay. As shown in Fig. 1c and d, ER stress enhanced the cell migration in resistant cells; however, migration was inhibited in Me1007 cells indicating that the adaptation to ER stress favoured cell migration in melanoma cells. To further confirm this finding, GFP-labelled Mel-RM, A375 and Me1007 cells (5 × 10^4^) were intravenously injected into nude mice who were subjected to bioluminescent imaging at 4 weeks after injection. The ER stress-resistant cells, Mel-RM and A375, exhibited a significant increase in lung metastatic abilities at approximately 4 weeks (Fig. 1e). Consistently, histological analyses indicated a remarkable increase in the number of metastatic lesions produced by Mel-RM and A375 cells compared with that produced by Me1007 cells (Fig. 1f-h).

### KLF4 is increased in melanoma cells under ER stress treatment

**Fig. 2:**
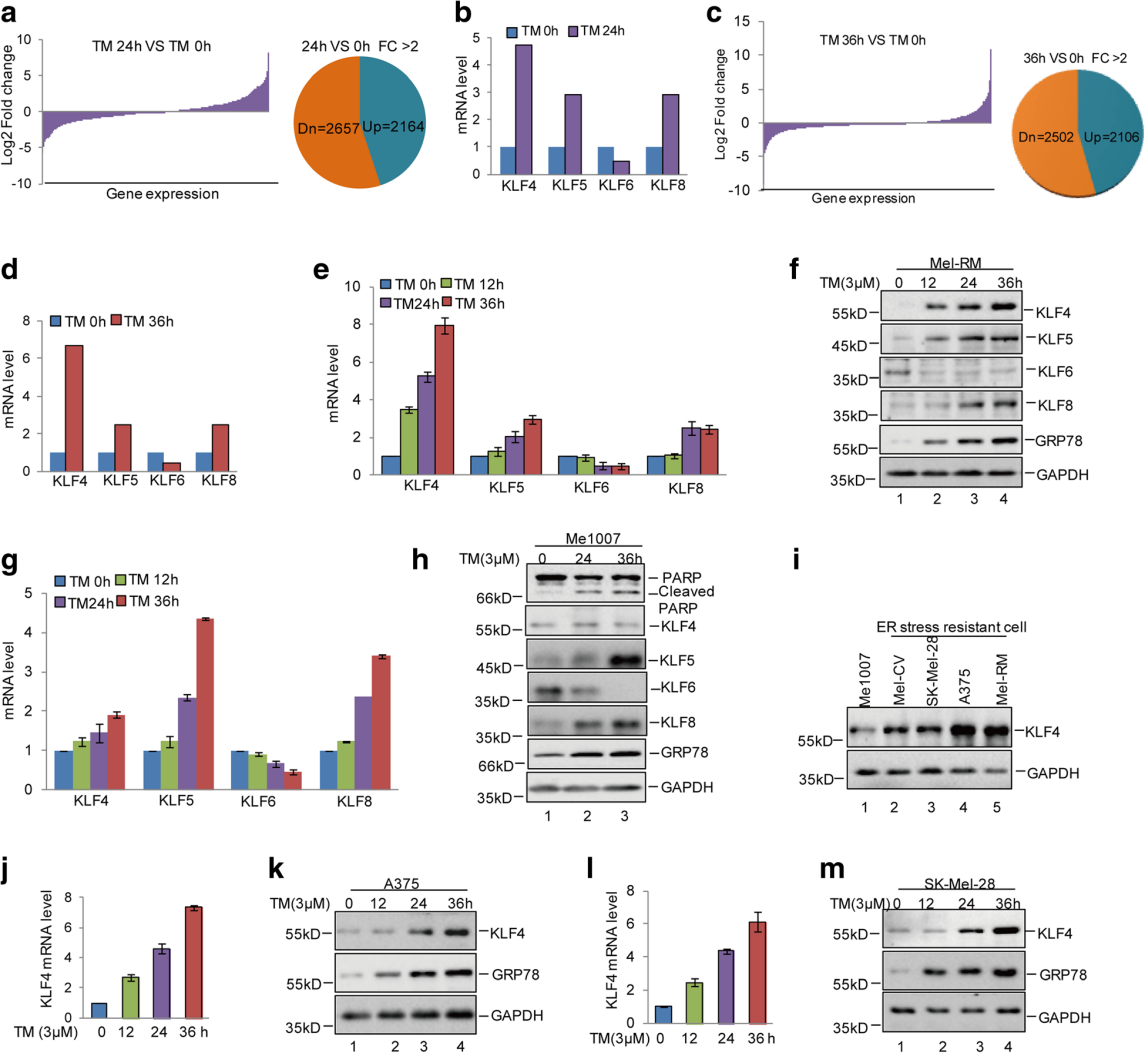
ER stress induced KLF4 expression. **a-d** Mel-RM cells were treated with 3 μM TM at the indicated times. Gene expression profiles were obtained by mRNA microarray analysis. **e-f** The expression levels of the KLF family genes KLF4, KLF5, KLF6 and KLF8 were analysed in Mel-RM cells with or without TM treatment by q-RT-PCR and western blot. **g-h** Me1007 cells were treated with 3 μM TM at the indicated times. The expression levels of the KLF family genes KLF4, KLF5, KLF6 and KLF8 were detected by q-RT-PCR and western blot. **i** The protein levels of KLF4 were analysed in Me1007, Mel-CV, SK-Mel-28, A375 and Mel-RM cells. **j-m** A375 and SK-Mel-28 cells were treated with 3 μM TM at the indicated times. The expression levels of KLF4 were detected by q-RT-PCR and western blot

To illuminate the mechanism underlying the regulation of ER stress resistance and cell metastasis, Mel-RM cells were treated with 3 μM TM for the indicated times, and then mRNA expression profiles were obtained by mRNA microarray analysis. The results revealed that more than 2100 upregulated and 2500 downregulated genes (FC > 2) were obtained in Mel-RM cells that were treated with 3 μM TM compared with that of control cells (Fig. 2a and c and Additional file 3: Table S1). Among the altered genes, we found that the KLF family genes KLF4, KLF5, KLF6 and KLF8 were changed in response to ER stress, and KLF4 was markedly upregulated under ER stress (Fig. 2b and d), which was confirmed by q-RT-PCR and western blot (Fig. 2e and f). To determine which gene may be involved in regulating ER stress resistance and cell metastasis, the expression levels of these genes were analysed in the ER stress non-resistant cell line Me1007 after 3 μM TM treatment. We found that compared with ER stress-resistant cells, the induction of KLF4 by ER stress disappeared in Me1007 cells, and the basic expression level of KLF4 was downregulated in Me1007 cells (Fig. 2g-i). However, the alteration of KLF5, KLF6 and KLF8 by ER stress were similar with the ER stress resistant cells. To further confirm this finding, the expression levels of KLF4 were first assessed in A375 and SK-Mel 28 cells using q-RT-PCR and western blot. Consistently, the protein and mRNA levels of KLF4 were elevated under 3 μM TM treatment (Fig. 2j-m). Then, we used 1 μM thapsigargin (TG), a Ca^2+^-ATPase inhibitor, to induce ER stress and the protein and mRNA levels of KLF4 were analysed using western blot and q-RT-PCR. Similar with 3 μM TM treatment, KLF4 was significantly increased in response to ER stress induced by 1 μM TG (Additional file 4: Figure S2a and b). Thus, these data implied that KLF4 was a ER stress-induced gene and may contribute ER stress resistance of melanoma.

### Increased KLF4 inhibits cell apoptosis and facilitates cell metastasis under ER stress

**Fig. 3:**
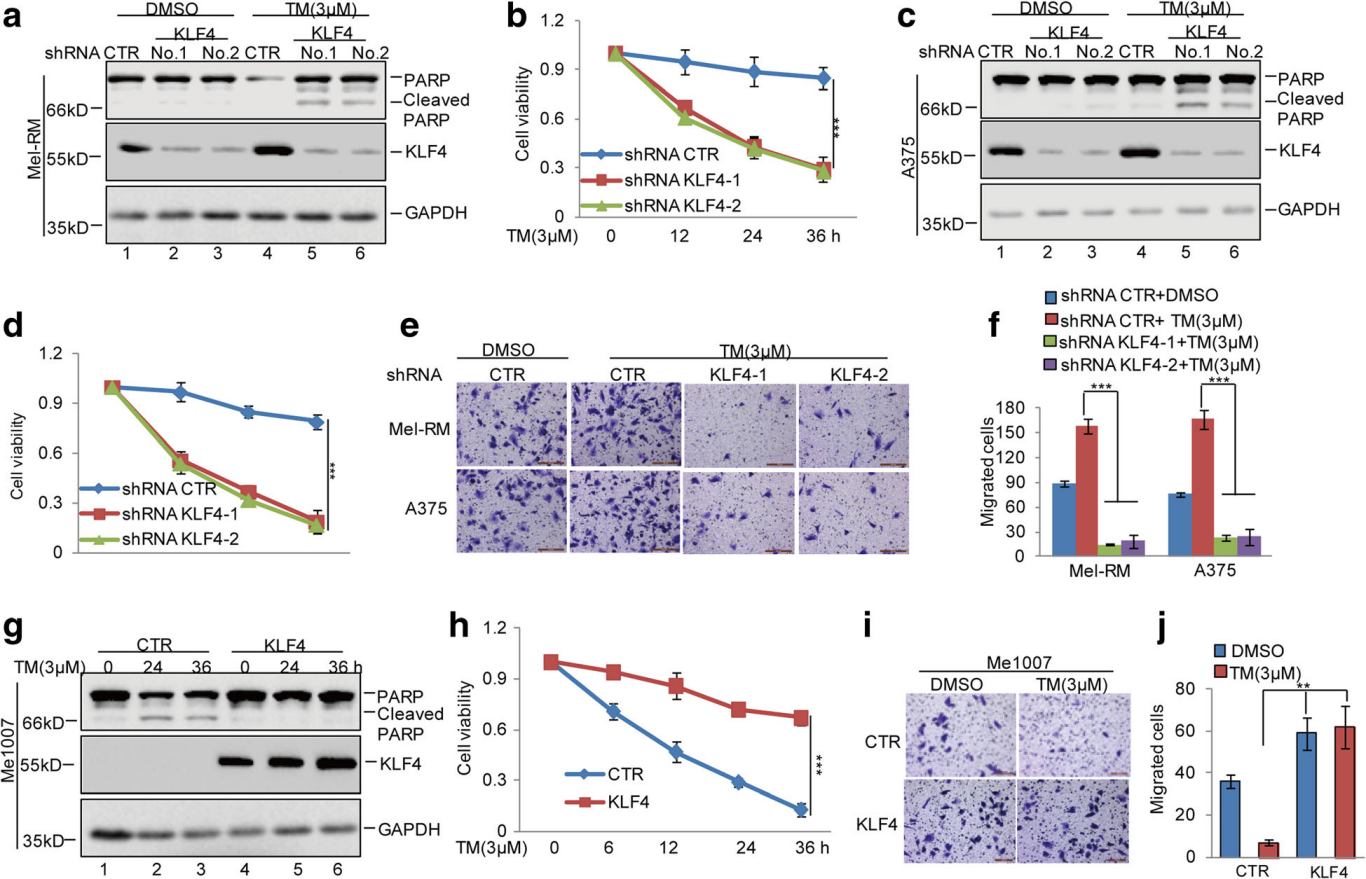
KLF4 inhibited ER stress-induced apoptosis and facilitated cell metastasis in melanoma cells. **a-d** KLF4 was stably knocked down in Mel-RM and A375 cells and then the cells were treated with 3 μM TM at the indicated times. Cell apoptosis was analysed by western blot and CCK8 assays. Data represent the mean ± SD of three independent experiments. ***p < 0.001 vs. control. **e-f** Cell migration was detected by a transwell assay. The data represent the means ± SD of three independent experiments; ***p < 0.001 vs. control. **g-h** KLF4 was stably overexpressed in Me1007 cells and then the cells were treated with 3 μM TM at the indicated times. Cell apoptosis was analysed by western blot and CCK8 assays. Data represent the mean ± SD of three independent experiments. ***p < 0.001 vs. control. **i-j** Cell migration was detected by a transwell assay. The data represent the means ± SD of three independent experiments; **p < 0.01 vs. control

Given that KLF4 enhanced melanoma adaptation to ER stress, we first knocked down KLF4,KLF5 and KLF8 expression using shRNAs in Mel-RM and A375 cells. Then, these cells were treated with 3 μM TM, and cell apoptosis was determined using western blot and the CCK-8 assay. As shown in Fig. 3a-d and Additional file 5: Figure S3a-b, the inhibition of KLF4 not KLF5 and KLF8 enhanced cell apoptosis and reduced cell viability. In subsequent transwell assays, the migratory capabilities of Mel-RM and A375 cells with KLF4 knockdown were apparently decreased in response to ER stress (Fig. 3e and f). To further verify this finding, we then overexpressed KLF4 in Me1007 cells. Consistent with data from the knockdown studies, Me1007 cells overexpressing KLF4 decreased cell apoptosis and enhanced cell migratory abilities under ER stress (Fig. 3g-j). Taken together, these data suggest that KLF4 plays an important role in regulating melanoma resistance to ER stress and facilitating cell metastasis.

### KLF4 upregulates NUCB2 expression

**Fig. 4:**
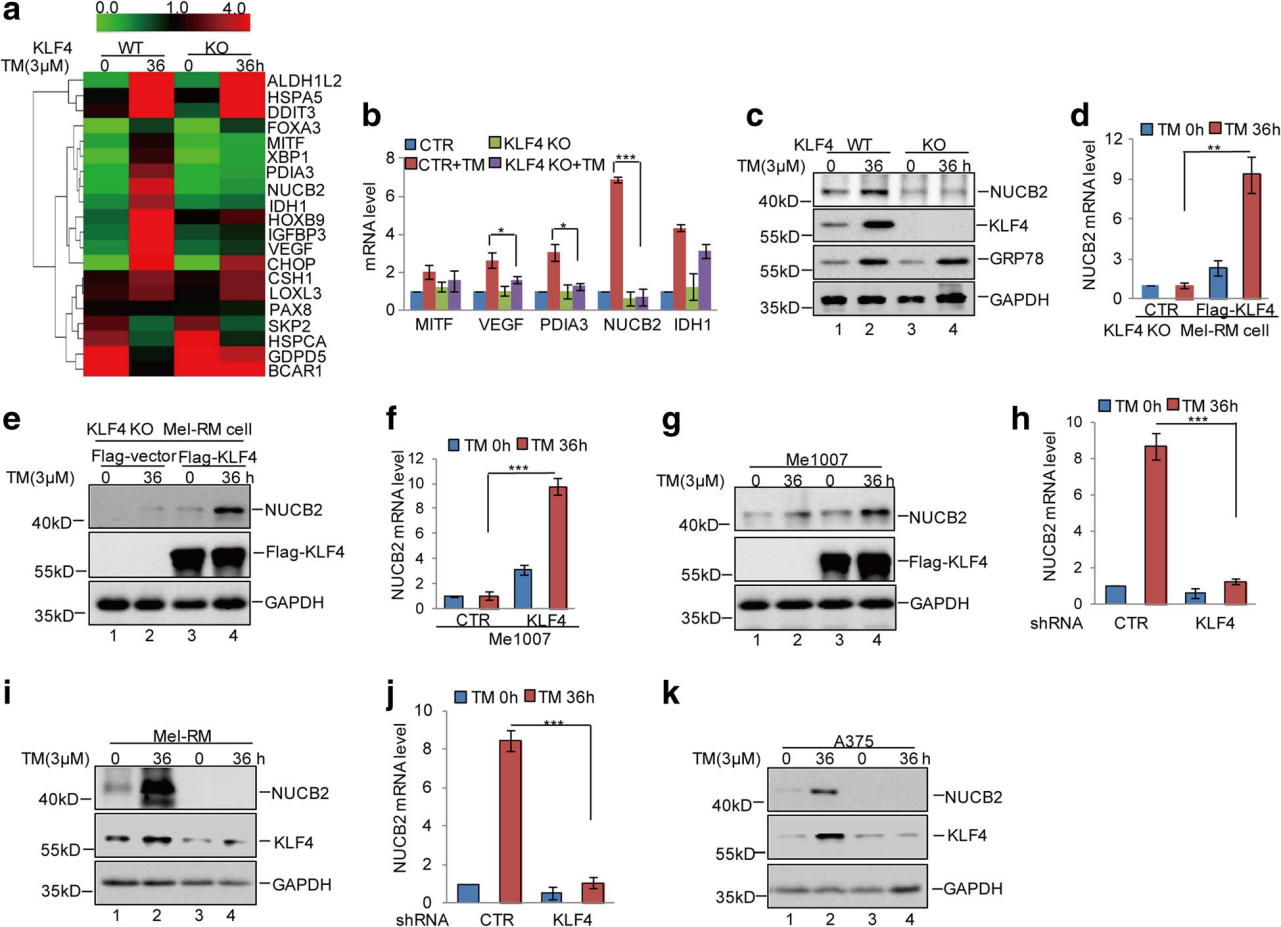
KLF4 upregulated NUCB2 expression in melanoma cells. **a** KLF4 WT or KO Mel-RM cells were treated with 3 μM TM at the indicated times. Gene expression profiles were obtained by RNA sequencing analysis. **b** The expression levels of MITF, VEGF, PDIA3, NUCB2 and IDH1 were analysed by q-RT-PCR. The data represent the means ± SD of three independent experiments; *p < 0.05, ***p < 0.001 vs. control. **c** The protein levels of KLF4 and NUCB2 were detected by western blot. **d-e** Flag-KLF4 and empty vector were individually transfected into KLF4 KO Mel-RM cells and the cells were treated with 3 μM TM at the indicated times. The expression levels of NUCB2 were analysed by q-RT-PCR and western blot assays. The data represent the means ± SD of three independent experiments; **p < 0.01 vs. control. **f-g** The Me1007 cells with or without KLF4 overexpression were treated using 3 μM TM at the indicated times. The expression levels of NUCB2 were analysed by q-RT-PCR and western blot assays. The data represent the means ± SD of three independent experiments; ***p < 0.001 vs. control. **h-k** Mel-RM and A375 cells with or without KLF4 knockdown were treated with 3 μM TM at the indicated times. The mRNA and protein levels of NUCB2 were detected by q-RT-PCR and western blot assays. Data represent the mean ± SD of three independent experiments. ***p < 0.001 vs. control

To uncover the molecular mechanism underlying the regulation of ER stress resistance by KLF4, we generated KLF4 knockout (KO) Mel-RM cells using CRISPR/Cas9 technology. Gene expression profiles in KLF4 WT and KO Mel-RM cells with or without 3 μM TM treatment were obtained using RNA sequencing analysis (Fig. 4a). Among the altered genes, we found that KLF4 KO significantly suppressed NUCB2 upregulation under ER stress, and the decrease was recovered by KLF4 overexpression (Fig. 4b-e). VEGF was used as a positive control, which was indicated to be a transcriptional gene of KLF4 [28] (Additional file 6: Figure S4a). Subsequently, the expression level of NUCB2 was analysed in Me1007 cells with or without KLF4 overexpression under 3 μM TM treatment. We found that NUCB2 was not induced by ER stress in Me1007 cells; however, in KLF4-overexpressing cells, the expression level of NUCB2 was increased, indicating that NUCB2 was mediated by KLF4 (Fig. 4f and g). To further confirm it, the expression level of NUCB2 was detected using q-RT-PCR and western blot in Mel-RM and A375 cells with or without KLF4 or KLF5 or KLF8 knockdown. Consistently, KLF4 not KLF5 and KLF8 knockdown suppressed the NUCB2 increase by ER stress (Fig. 4h-k and Additional file 6: Figure S4b-e). Collectively, these data validate that KLF4 upregulated NUCB2 expression in response to ER stress.

### KLF4 directly binds to the promoter of NUCB2

**Fig. 5:**
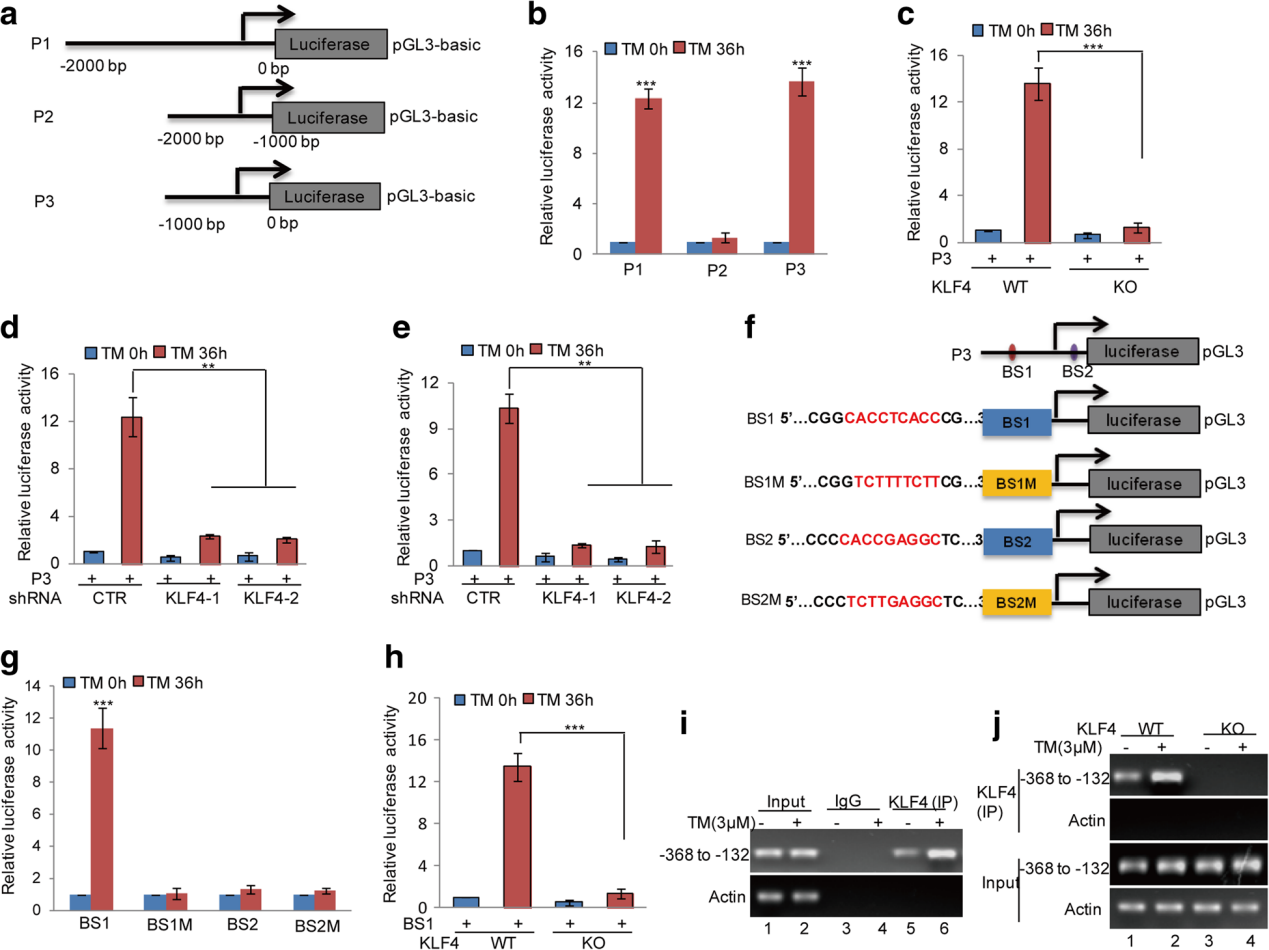
KLF4 was bound to the promoter of NUCB2 in melanoma cells. **a** Schematic illustration of pGL3-based reported constructs were used in luciferase assays to examine the transcriptional activity of NUCB2. **b** The promoters of NUCB2 named P1, P2 and P3 were individually transfected into Mel-RM cells with or without TM treatment. The luciferase activity was measured. The data represent the means ± SD of three independent experiments; ***p < 0.001 vs. control. **c** P3 was transfected into Mel-RM cells with or without KLF4 knockout. The cells were treated with 3 μM TM. The luciferase activity of NUCB2 promoter was measured by the luciferase reporter assay. The data represent the means ± SD of three independent experiments; ***p < 0.001 vs. control. **d-e** P3 was transfected into Mel-RM and A375 cells with or without KLF4 knockdown. The cells were treated with 3 μM TM. The luciferase activity of NUCB2 promoter was measured by the luciferase reporter assay. The data represent the means ± SD of three independent experiments; **p < 0.01 vs. control. **f** The potential KLF4-binding sites were inspected by JASPAR. A schematic illustration of KLF4 wild type binding site 1 (BS1), binding site 2 (BS2) and the matching mutant BS1M, BS2M that were used in the luciferase assays is shown. **g** BS1, BS1M, BS2 and BS2M were individually transfected into Mel-RM cells with or without 3 μM TM treatment. The luciferase activity was measured by the luciferase reporter assay. The data represent the means ± SD of three independent experiments; ***p < 0.001 vs. control. **h** BS1 was transfected into Mel-RM with or without KLF4 knockout. The cells were treatment with 3 μM TM. The luciferase activity was measured by the luciferase reporter assay. The data represent the means ± SD of three independent experiments; ***p < 0.001 vs. control. **i-j** ChIP analysis showed the binding of KLF4 to the promoter of NUCB2 in KLF4 WT or KO Mel-RM cells in response to 3 μM TM treatment. An isotype-matched IgG was used as a negative control

To identify the KLF4 binding regions on the NUCB2 promoter, we first cloned the upstream sequence of NUCB2 and different truncations by PCR. Then, we inserted them into the pGL3-based luciferase reporter plasmids that were named P1-P3 (Fig. 5a). We subsequently transfected them into Mel-RM cells with or without 3 μM TM treatment. As shown in Fig. 5b, the luciferase activities of P1 and P3 were increased in Mel-RM cells under ER stress treatment; however, the increase was abolished when P2 was transfected, indicating that the region (− 1000 to 0 bp) was a key region for the promotion of NUCB2 under ER stress. To further verify whether the region was essential for KLF4, the truncation P3 was transfected into Mel-RM cells with or without KLF4 knockout. We found that the loss of KLF4 abolished the increase of the luciferase activity of P3 by ER stress (Fig. 5c). Similar results were gained in Mel-RM and A375 cells with or without KLF4 knockdown (Fig. 5d-e).

KLF4 is a zinc finger-type transcription factor that usually binds to the GC-rich element of the promoters [29]. To seek the potential KLF4 binding sites, we inspected the sequence of P3 using JASPAR software and found two putative KLF4-binding sites on the NUCB2 promoter. To verify that these potential KLF4-binding sites are indeed responsive to KLF4, a series of pGL3-based luciferase reporter plasmids named BS1, BS1M, BS2 and BS2M were generated (Fig. 5f). These plasmids were individually transfected into Mel-RM cells with or without 3 μM TM treatment. As shown in Fig. 5g and h, the luciferase activity of BS1, but not BS1M, BS2, and BS2M, was significantly increase in KLF4 WT Mel-RM cells under ER stress, and the increase disappeared when KLF4 was knocked out, indicating that the first binding site was a positive KLF4 binding site on the NUCB2 promoter. In addition, the subsequent chromatin immunoprecipitation (ChIP) assays showed that the chromatin fragments, corresponding to the putative KLF4-binding sites, were specifically present in anti-KLF4 immunoprecipitates from Mel-RM cells, and the bond was increased under ER stress, which was diminished by KLF4 knockout (Fig. 5i-j).

### KLF4 elevates the melanoma adaptation to ER stress and metastasis by regulating NUCB2 in vitro and in vivo

**Fig. 6:**
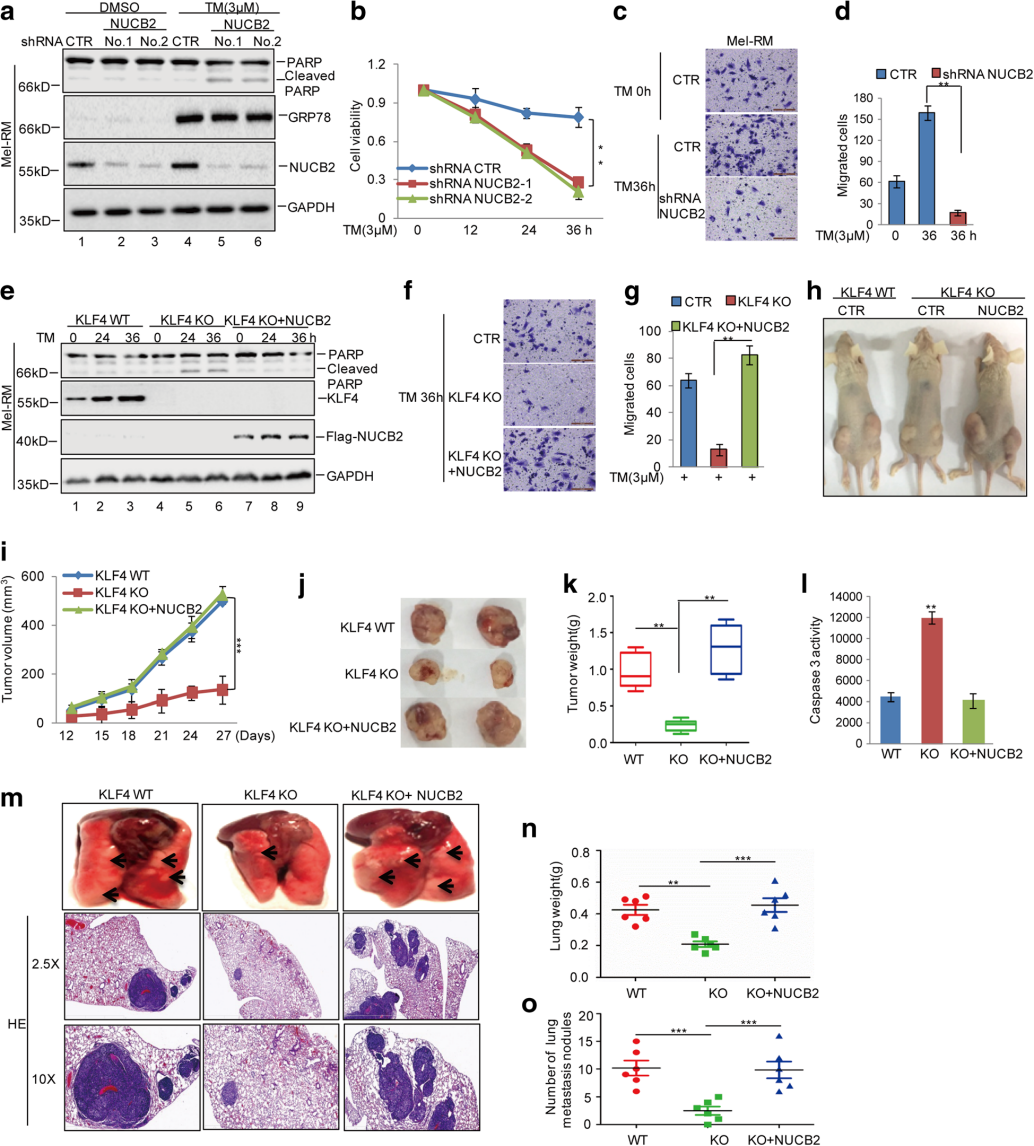
KLF4 elevated the adaptation to ER stress and cell metastasis via regulating NUCB2 expression. **a-d** Mel-RM cells with or without NUCB2 knockdown were treated with 3 μM TM. The cell apoptosis was detected by western blot and CCK-8 assays. Cell migration was analysed using a transwell assay. The data represent the means ± SD of three independent experiments; **p < 0.01 vs. control. **e-g** Flag-NUCB2 and empty vector were individually transfected into Mel-RM cells with or without KLF4 knockout. The cells were treated with 3 μM TM at the indicated times. Cell apoptosis was detected by a western blot assay and cell migration was analysed using a transwell assay. The data represent the means ± SD of three independent experiments; **p < 0.01 vs. control. **h-l** KLF4 WT or KO Mel-RM cells with or without NUCB2 expression were subcutaneously injected into nude mice (*n* = 6 in each group) for tumour formation (1 × 10^6^ cells per mouse, 4 weeks). Representative bright-field imaging of the tumours in the mice implanted the indicated cells. After 4 weeks, mice receiving transplants of indicated cells were sacrificed. The tumour volume and weight were calculated. Caspase-3 activity was measured by a luciferase activity assay; **p < 0.01 vs. control. **m-o** The cells as indicated (5 × 10^4^) were injected intravenously into nude mice (*n* = 6 in each group). Representative images of lung and HE pictures were shown (**m**), and the weight and metastasis nodule of the lung in each group were calculated (**g**-**h**); **p < 0.01, ***p < 0.001 vs. control

To determine whether the promotion of KLF4 on the adaptation to ER stress and cell metastasis was dependent on NUCB2, we first knocked down NUCB2 in Mel-RM cells, and then the cells were treated with 3 μM TM. Compared with the control cells, deficiency in NUCB2 promoted ER stress-induced apoptosis and decreased cell migration, which was consistent with the role of KLF4 in melanoma cells (Fig. 6a-b). Whereafter, we overexpressed NUCB2 into KLF4 KO Mel-RM cells and found that the effects of KLF4 depletion on melanoma adaptation to ER stress and metastasis were reversed by NUCB2 overexpression (Fig. 6e-g).

To further identify the effects of KLF4 on the adaptation to ER stress and cell metastasis by regulating NUCB2 in vivo, NUCB2 was overexpressed into KLF4 KO Mel-RM cells, and the expression levels of KLF4 and NUCB2 were detected using western blot (Additional file 7: Figure S5). First, the cells were subcutaneously injected into nude mice. Compared with control cells, the loss of KLF4 decreased tumour growth and increased cell apoptosis; however, the phenotypes were reversed by NUCB2 overexpression (Fig. 6h-l). The cells were then injected intravenously into the nude mice, and the migratory abilities of these cells were assessed in vivo. As shown in Fig. 6m-o, the deficiency of KLF4 decreased lung metastatic abilities of melanoma cells, which was reversed by NUCB2 overexpression. Taken together, these data indicate that the effects of KLF4 on melanoma adaptation to ER stress and metastasis were dependent on NUCB2.

### The expression of KLF4 is increased in melanoma tissues and is associated with NUCB2 expression

**Fig. 7:**
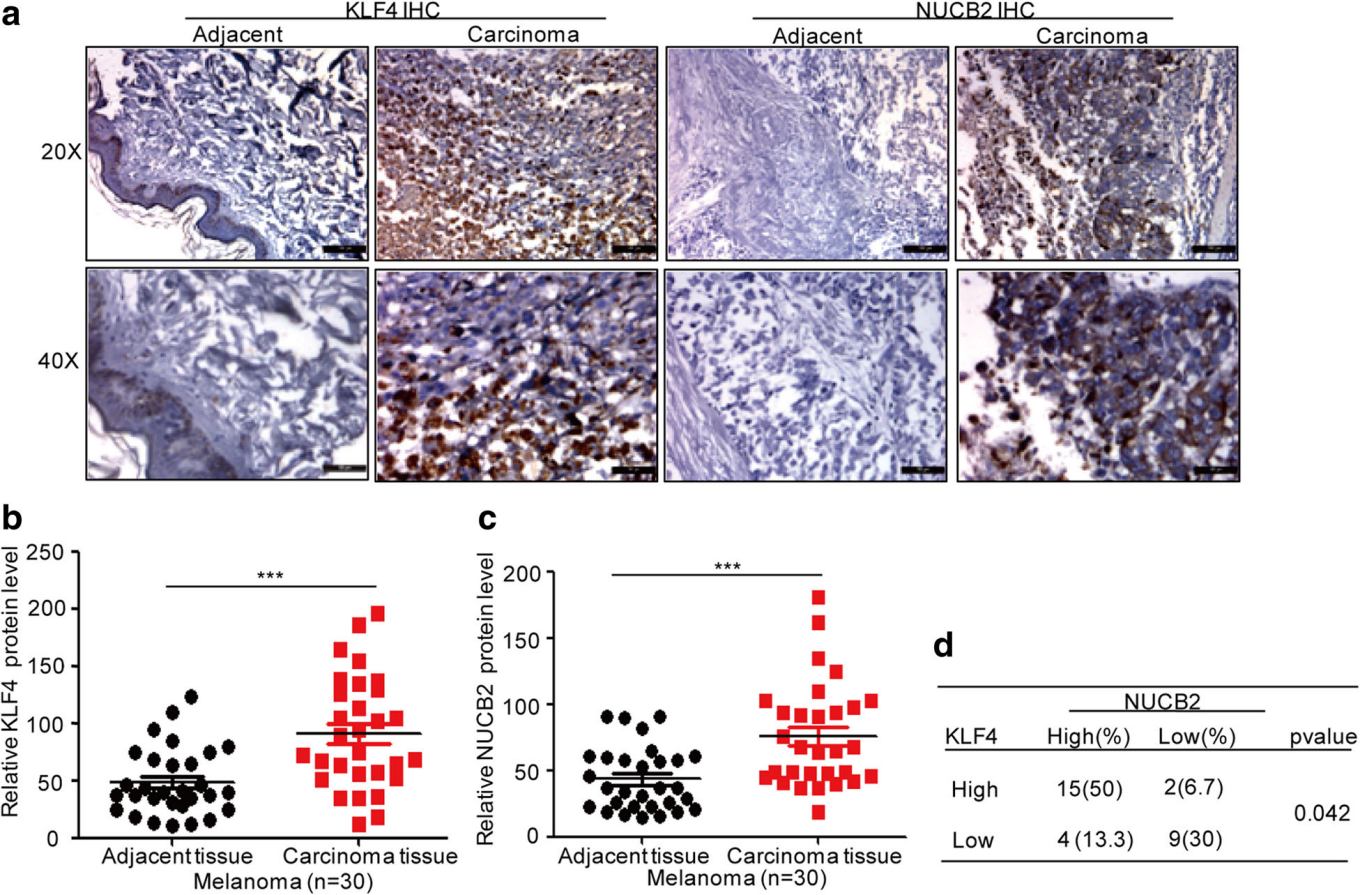
The correlation between KLF4 and NUCB2 was analysed in melanoma tissues. **a-c** The protein levels of KLF4 and NUCB2 in melanoma tissues and adjacent normal tissues (*n* = 30) were detected by immunohistochemical staining; ***p < 0.001 vs. control. **d** The correlation between KLF4 and NUCB2 was analysed in melanoma tissues

To further confirm the biological importance of the KLF4-NUCB2 axis in melanoma, the protein levels of KLF4 and NUCB2 in melanoma tissues and normal tissues were analysed using immunohistochemical staining (Fig. 7a). We found that the relative expression levels of KLF4 and NUCB2 were significantly increased in melanoma tissues, and the protein level of KLF4 was positively correlated with NUCB2 in melanoma tissues (Fig. 7b-d).

## Discussion

In this study, we investigated the role of ER stress in melanoma cell metastasis and found that adaptation to ER stress favoured cell metastasis, otherwise, decreasing cell metastasis. Further mechanistic studies indicated that KLF4 induced by ER stress inhibited ER stress-induced apoptosis and promoted cell metastasis by transcriptionally upregulating NUCB2 expression in vivo and in vitro. Thus, our data indicated a mechanism in which adaptation to ER stress plays a key role in accelerating melanoma cell metastasis.

ER stress is an important biologic process that is usually induced by physiological and pathological conditions such as nutrient deprivation, hypoxia, ER Ca^2+^ depletion, impaired glycosylation or disulfide bond formation, oxidative stress and viral or bacterial infection [16]. Adaptation to ER stress has been indicated to play an essential role in melanoma, which not only renders them resistant to various therapeutic agents but also favours the escape of melanoma cells from anti-tumour immunity [30–33]. Similarly, our data indicated that melanoma adaptation to ER stress was an important cause for promoting cell metastasis. We also found that KLF4 induced by ER stress promoted melanoma adaptation to ER stress and cell metastasis.

KLF4 is a zinc finger-type transcription factor that modulates diverse and essential functions in multiple cellular processes, including proliferation, differentiation, migration, inflammation and pluripotency [34, 35]. In tumours, KLF4 acts as an oncogene or a tumour suppressor depending on the types of cancers [36–38]. Recent studies indicated that KLF4 was increased in melanoma and enhanced tumour growth [39]. Consistently, our findings provided a series of evidence supporting the oncogenic role of KLF4 in melanoma adaptation to ER stress. First, KLF4 ablation dramatically reduced melanoma adaption to ER stress and inhibited cell metastasis in vitro and in vivo. Second, exogenous expression of KLF4 in ER stress non-resistant cells suppressed cell apoptosis and promoted cell metastasis. Third, the expression levels of KLF4 were increased in melanoma tissues and KLF4 was induced by ER stress in a transcriptional manner. Although KLF4 is modulated by a variety of environmental signals, including DNA damage, inflammation and oxidative stress, the molecular mechanism of the KLF4 increase under ER stress is still unknown. Recent study indicated that E2F1 was a potential transcription factor in melanoma. However, we need further study to investigate whether E2F1 involved in the KLF4 increase under ER stress.

KLF4 can both activate and repress transcription, depending on the contents of target promoters and its interacting partners [29, 35]. To seek the targeted genes of KLF4 under ER stress, the gene expression profiles in KLF4 WT and KO Mel-RM cells with or without TM treatment were obtained by RNA sequencing analysis. Among the altered genes, we found that KLF4 KO decreased NUCB2 expression in response to ER stress.

NUCB2, a neuropeptide, is a metablic factor that plays an important role in food intake and energy homeostasis [40]. An increasing number of studies indicated that NUCB2 acts as a tumour promoter enhancing tumourigenesis and metastasis in breast cancer and renal cell carcinoma (RCC) [41–44]. In our study, we uncovered the oncogene role of NUCB2 in melanoma and found that NUCB2 was induced by ER stress for the first time. Furthermore, our data also showed that elevated NUCB2 plays an important role in inhibiting ER stress-induced apoptosis and promoting cell metastasis in melanoma, and the expression level of NUCB2 was increased in melanoma tissues. Although the role of NUCB2 was indicated in our study, the downstream pathway of NUCB2 in melanoma will be explored in the future. In brief, our data showed that adaptation to ER stress is essential for promoting melanoma metastasis and provided strong evidence that the KLF4-NUCB2 pathway contributes to melanoma metastasis under ER stress by elevating melanoma ER stress resistance.

## Conclusions

In summary, our study indicates that KLF4 was an important regulator in melanoma adaptation to ER stress and cell metastasis. We found that KLF4 induced by ER stress could directly bind to the promoter of NUCB2 and promote NUCB2 expression, leading to the increase in melanoma ER stress resistance and cell metastasis. Thus, our data revealed that the KLF4-NUCB2 pathway plays an important role in melanoma ER stress resistance and cell metastasis, and KLF4 may be a promising specific target for melanoma therapy.

## Additional files

Additional file 1: **Table S2.** Clinical information of Patients with melanoma. (XLSX 11 kb)

Additional file 2: **Figure S1.** Me1007, Mel-CV, SK-Mel-28, A375 and Mel-RM cells were treated with 3 μM TM. Cell viability was measured by the CCK-8 assay. The data represent the means ± SD of three independent experiments. ***p < 0.001 vs. control. (TIF 52 kb)

Additional file 3: **Table S1.** Differentially expressed genes. (XLS 4331 kb)

Additional file 4: **Figure S2.** (a-b) Mel-RM and A375 cells were treated with 1 μM TG for the indicated times. The expression levels of KLF4 were detected using western blot and q-RT-PCR. The data represent the means ± SD of three independent experiments. (TIF 196 kb)

Additional file 5: **Figure S3.** (a-b) Mel-RM with or without KLF5 or KLF8 knockdown were treated with 3 μM TM. Cell lysates were then subjected to western blot analysis using the indicated antibodies. (TIF 192 kb)

Additional file 6: **Figure S4.** (a) the mRNA levels of VEGF were analysed by q-RT-PCR in KLF4 WT, KLF4 KO or KLF4 KO + Flag-KLF4 Mel-RM cells with or without 3 μM TM treatment for the indicated times. The data represent the means ± SD of three independent experiments. **p < 0.01, ***p < 0.001 vs. control. (b-e) The expression levels of NUCB2 were detected by western blot and q-RT-PCR assays in Mel-RM cells with or without KLF5 or KLF8 knockdown under TM treatment. The data represent the means ± SD of three independent experiments. ***p < 0.001 vs. control. (TIF 252 kb)

Additional file 7: **Figure S5.** NUCB2 was transfected in a stable manner into Mel-RM cells with or without KLF4 knockout. Cell lysates were then subjected to western blot analysis using the indicated antibodies. (TIF 69 kb)

## Abbreviations

CCK-8: Cell Counting Kit-8
ChIP: Chromatin immunoprecipitation
CTLA-4: Cytotoxic T-lymphocyte-associated protein 4
ER stress: Endoplasmic reticulum stress
HOXB9: Homeobox protein Hox-B9
KLF4: Kruppel-like factor 4
KO: Knockout
MEK: Mitogen-activated protein extracellular kinase
NUCB2: Nucleobindin 2
PD1: Programmed cell death protein 1
q-RT-PCR: Quantitative real-time polymerase chain reaction assay
RCC: Renal cell carcinoma
RIPK1: Receptor-interacting serine/threonine-protein kinase 1
TM: Tunicamycin
TMA: Human tissue microarray analysis
