# Correction to: Regulation of the adaptation to ER stress by KLF4 facilitates melanoma cell metastasis via upregulating NUCB2 expression

**DOI:** 10.1186/s13046-021-02181-5

**Published:** 2021-12-07

**Authors:** Dongmei Zhang, Jingrong Lin, Yulin Chao, Lu Zhang, Lei Jin, Na Li, Ruiping He, Binbin Ma, Wenzhi Zhao, Chuanchun Han

**Affiliations:** 1 Institute of Cancer Stem Cell, Dalian Medical University, Dalian, 116044 China; 2 Department of Orthopedics, Second Affiliated Hospital, Dalian Medical University, Dalian, 116044 China; 3 Department of Physiology, College of Basic Medical Sciences, Dalian Medical University, Dalian, 116044 China; 4 Department of Dermatology, the First Affiliated Hospital, Dalian Medical University, Liaoning, 116027 China

**Correction to: J Exp Clin Cancer Res 37, 176 (2018).**

**https://doi.org/10.1186/s13046-018-0842-z**

Following publication of the original article [1], the authors identified some minor errors in Figs. 1, 3, 4 and 6, specifically:

**Fig. 1:**
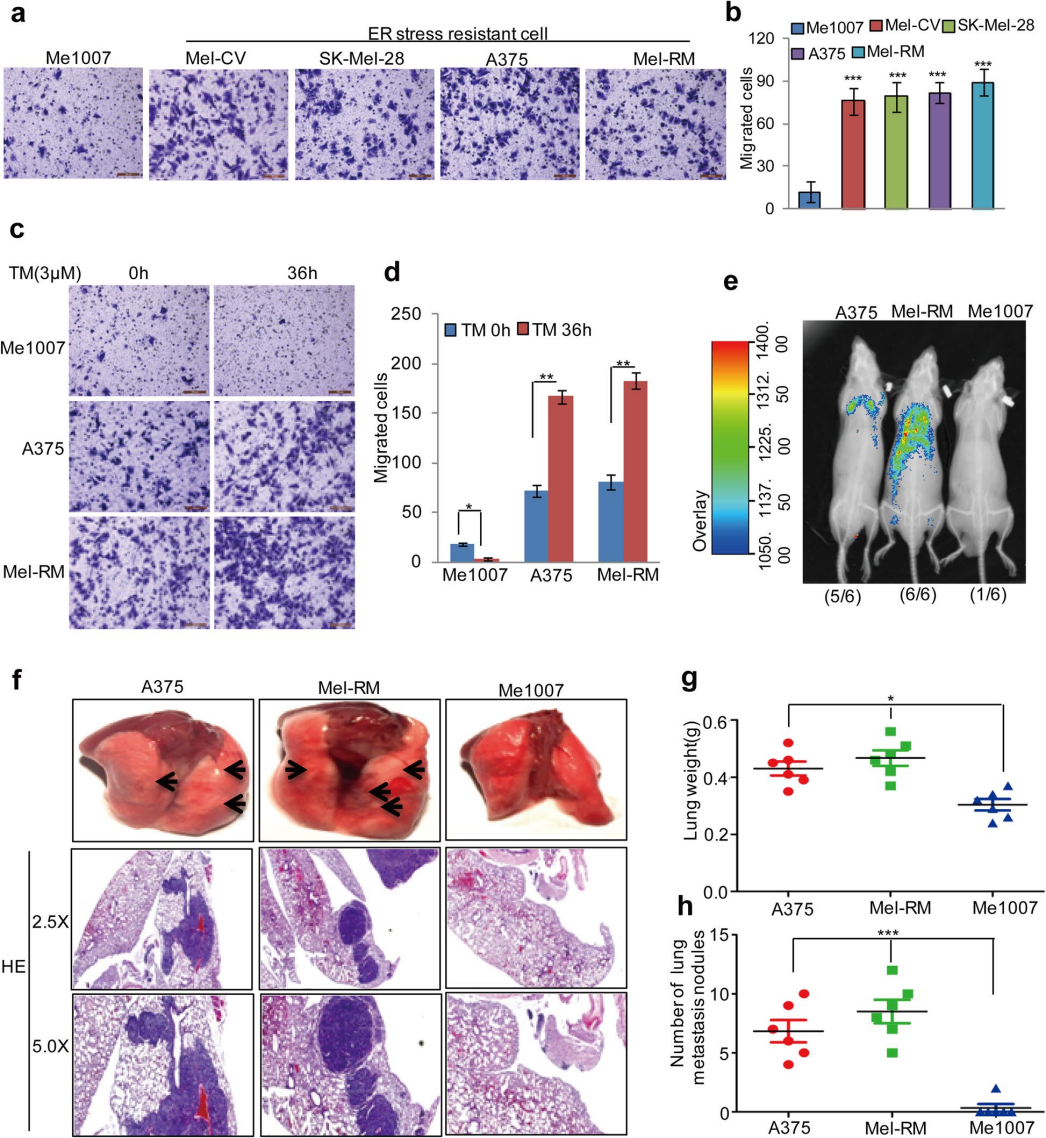
ER stress-resistant cells exhibited more migratory phenotype. **a-b** The migration abilities of the melanoma cells were detected by a transwell assay. The data represent the means ± SD of three independent experiments; ***p < 0.001 vs. control. **c-d** Me1007, A375 and Mel-RM cells were treated with 3 μM TM as indicated. Cell migration was analysed by a transwell assay. The data represent the means ± SD of three independent experiments; *p < 0.05,**p < 0.01 vs. control. **e** GFP-labelled Mel-RM, A375 and Me1007 cells (5 × 104) were injected intravenously into nude mice (*n* = 6 in one group). After 4 weeks, the mice were subjected to bioluminescent imaging. **f-h** Representative images of the lung and HE pictures are shown (**f**), the weight and metastasis nodule in each group were calculated (**g-h**); *p < 0.05, ***p < 0.001 vs. control

**Fig. 3:**
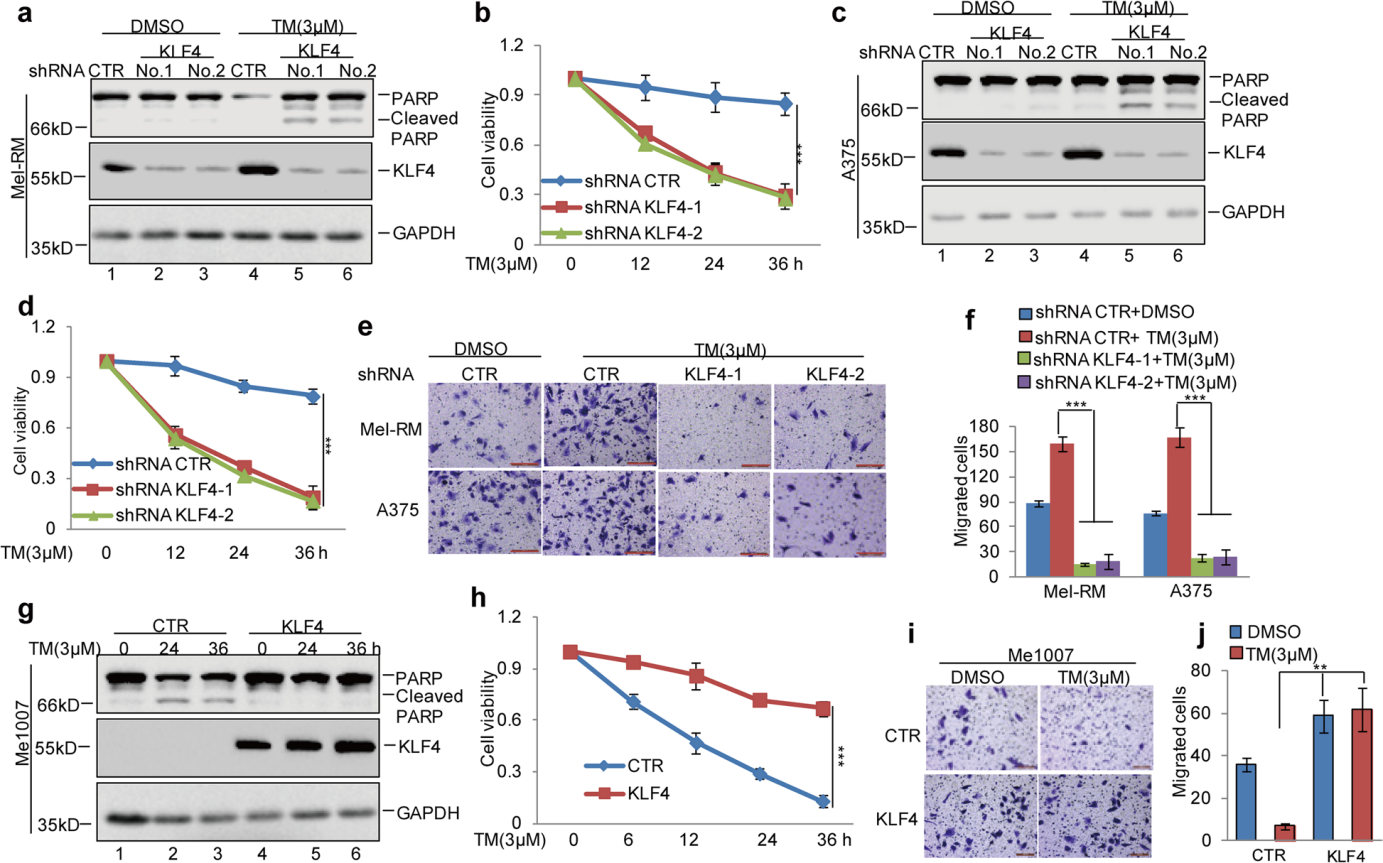
KLF4 inhibited ER stress-induced apoptosis and facilitated cell metastasis in melanoma cells. **a-d** KLF4 was stably knocked down in Mel-RM and A375 cells and then the cells were treated with 3 μM TM at the indicated times. Cell apoptosis was analysed by western blot and CCK8 assays. Data represent the mean ± SD of three independent experiments. ***p < 0.001 vs. control. **e-f** Cell migration was detected by a transwell assay. The data represent the means ± SD of three independent experiments; ***p < 0.001 vs. control. **g-h** KLF4 was stably overexpressed in Me1007 cells and then the cells were treated with 3 μM TM at the indicated times. Cell apoptosis was analysed by western blot and CCK8 assays. Data represent the mean ± SD of three independent experiments. ***p < 0.001 vs. control. **i-j** Cell migration was detected by a transwell assay. The data represent the means ± SD of three independent experiments; **p < 0.01 vs. control

**Fig. 4:**
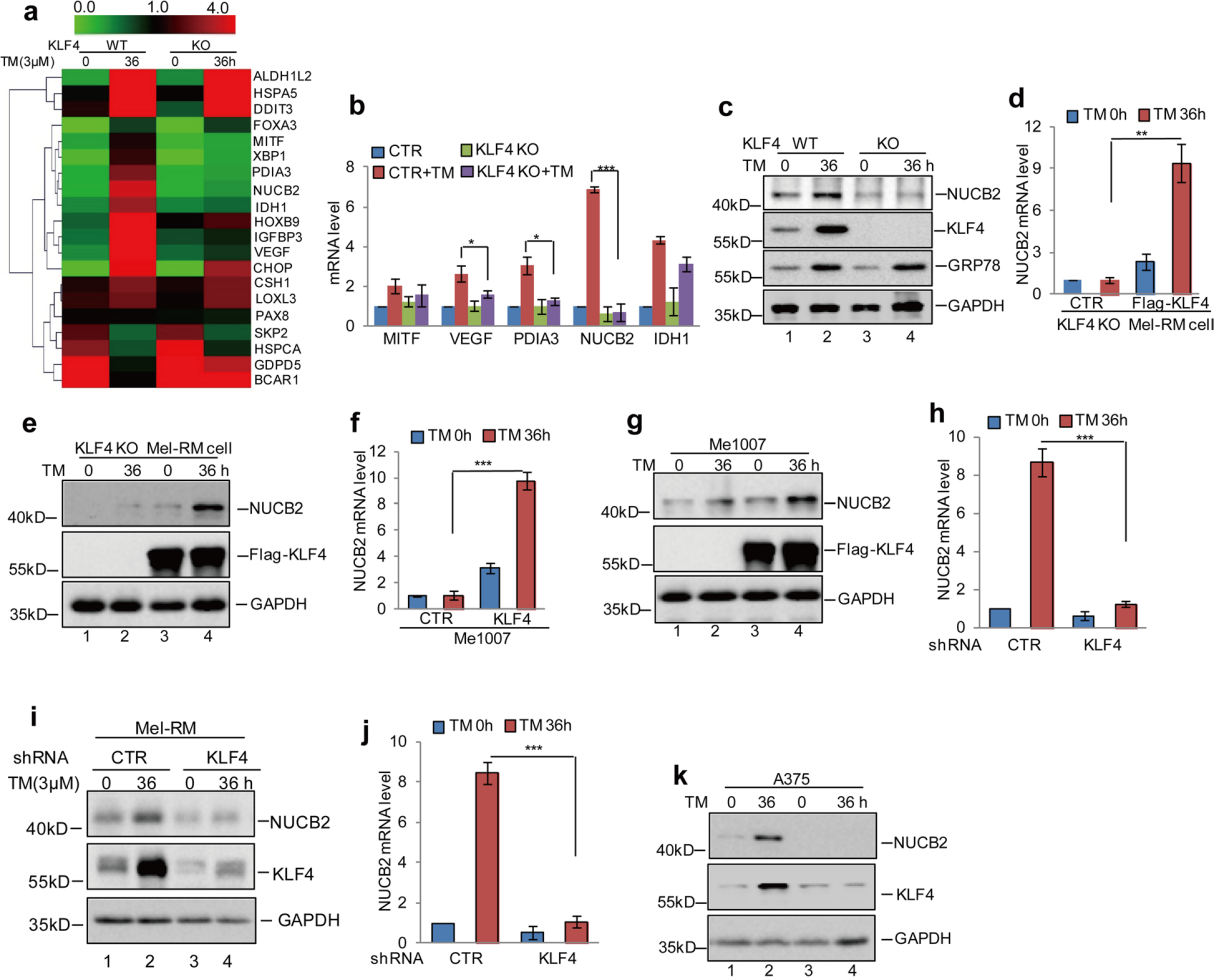
KLF4 upregulated NUCB2 expression in melanoma cells. **a** KLF4 WT or KO Mel-RM cells were treated with 3 μM TM at the indicated times. Gene expression profiles were obtained by RNA sequencing analysis. **b** The expression levels of MITF, VEGF, PDIA3, NUCB2 and IDH1 were analysed by q-RT-PCR. The data represent the means ± SD of three independent experiments; *p < 0.05, ***p < 0.001 vs. control. **c** The protein levels of KLF4 and NUCB2 were detected by western blot. **d-e** Flag-KLF4 and empty vector were individually transfected into KLF4 KO Mel-RM cells and the cells were treated with 3 μM TM at the indicated times. The expression levels of NUCB2 were analysed by q-RT-PCR and western blot assays. The data represent the means ± SD of three independent experiments; **p < 0.01 vs. control. **f-g** The Me1007 cells with or without KLF4 overexpression were treated using 3 μM TM at the indicated times. The expression levels of NUCB2 were analysed by q-RT-PCR and western blot assays. The data represent the means ± SD of three independent experiments; ***p < 0.001 vs. control. **h-k** Mel-RM and A375 cells with or without KLF4 knockdown were treated with 3 μM TM at the indicated times. The mRNA and protein levels of NUCB2 were detected by q-RT-PCR and western blot assays. Data represent the mean ± SD of three independent experiments. ***p < 0.001 vs. control

**Fig. 6:**
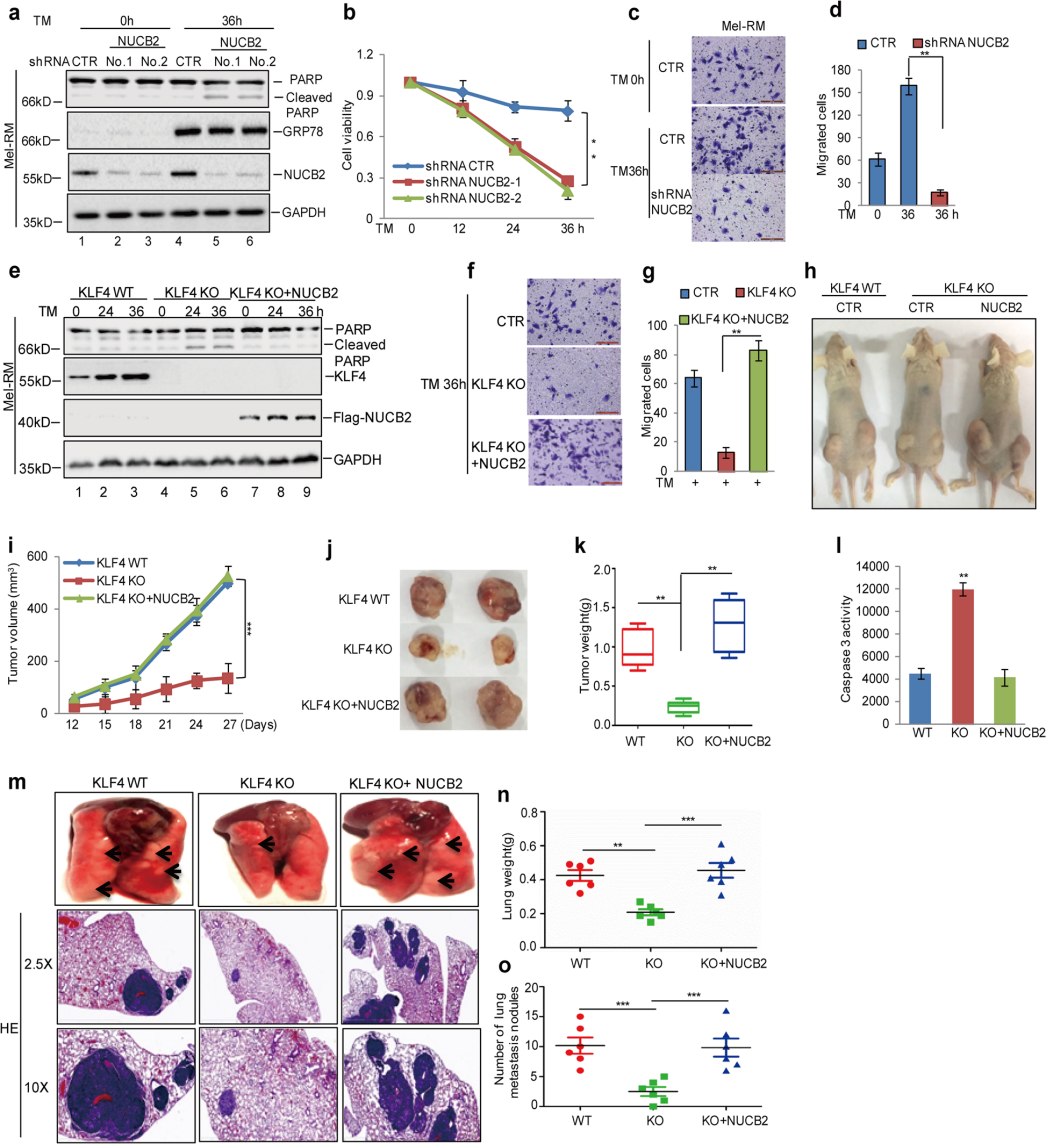
KLF4 elevated the adaptation to ER stress and cell metastasis via regulating NUCB2 expression. **a-d** Mel-RM cells with or without NUCB2 knockdown were treated with 3 μM TM. The cell apoptosis was detected by western blot and CCK-8 assays. Cell migration was analysed using a transwell assay. The data represent the means ± SD of three independent experiments; **p < 0.01 vs. control. **e-g** Flag-NUCB2 and empty vector were individually transfected into Mel-RM cells with or without KLF4 knockout. The cells were treated with 3 μM TM at the indicated times. Cell apoptosis was detected by a western blot assay and cell migration was analysed using a transwell assay. The data represent the means ± SD of three independent experiments; **p < 0.01 vs. control. **h-l** KLF4 WT or KO Mel-RM cells with or without NUCB2 expression were subcutaneously injected into nude mice (*n* = 6 in each group) for tumour formation (1 × 106 cells per mouse, 4 weeks). Representative bright-field imaging of the tumours in the mice implanted the indicated cells. After 4 weeks, mice receiving transplants of indicated cells were sacrificed. The tumour volume and weight were calculated. Caspase-3 activity was measured by a luciferase activity assay; **p < 0.01 vs. control. **m-o** The cells as indicated (5 × 104) were injected intravenously into nude mice (n = 6 in each group). Representative images of lung and HE pictures were shown (**m**), and the weight and metastasis nodule of the lung in each group were calculated (g-h); **p < 0.01, ***p < 0.001 vs. control

Figure 1a: incorrect image was used for the migration image of Mel-CV.

Figure 3e: incorrect images were used for the migration images of Mel-RM shRNA CTR and A375 shKLF4–2.

Figure 4i: the KLF4 western blot band of shRNA KLF4 TM 36 h group was incomplete.

Figure 4i: incorrect image was used for the band of NUCB2.

Figure 6f: incorrect image was used for the migration image of KLF4 KO + NUCB2 TM 36 h group.

The authors provided the Journal with the original data files. The corrected figures are given here. The corrections do not have any effect on the final conclusions of the paper. The original article has been corrected.

## References

[1] Zhang D, Lin J, Chao Y (2018). Regulation of the adaptation to ER stress by KLF4 facilitates melanoma cell metastasis via upregulating NUCB2 expression. J Exp Clin Cancer Res.

