# Supplementary figures and images for: Regulation of the adaptation to ER stress by KLF4 facilitates melanoma cell metastasis via upregulating NUCB2 expression
Source: J Exp Clin Cancer Res. 2018 Jul 28;37:176. doi: 10.1186/s13046-018-0842-z (PMC6064624; doi:10.1186/s13046-018-0842-z)

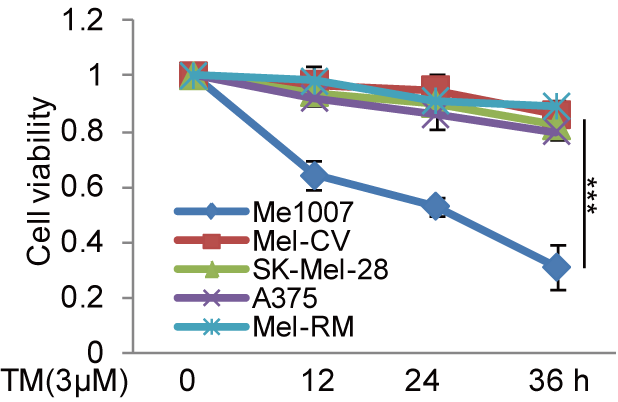

Supplement: Supplementary file 2 — Figure S1. Me1007, Mel-CV, SK-Mel-28, A375 and Mel-RM cells were treated with 3 μM TM. Cell viability was measured by the CCK-8 assay. The data represent the means ± SD of three independent experiments. ***p < 0.001 vs. control. (TIF 52 kb) [file 13046_2018_842_MOESM2_ESM.tif]

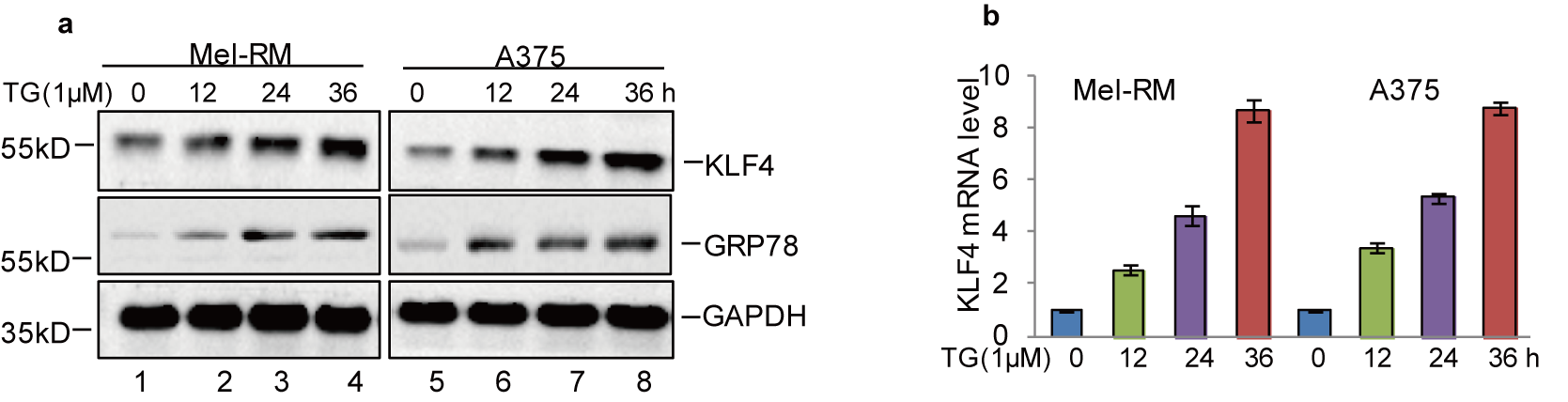

Supplement: Supplementary file 4 — Figure S2. (a-b) Mel-RM and A375 cells were treated with 1 μM TG for the indicated times. The expression levels of KLF4 were detected using western blot and q-RT-PCR. The data represent the means ± SD of three independent experiments. (TIF 196 kb) [file 13046_2018_842_MOESM4_ESM.tif]

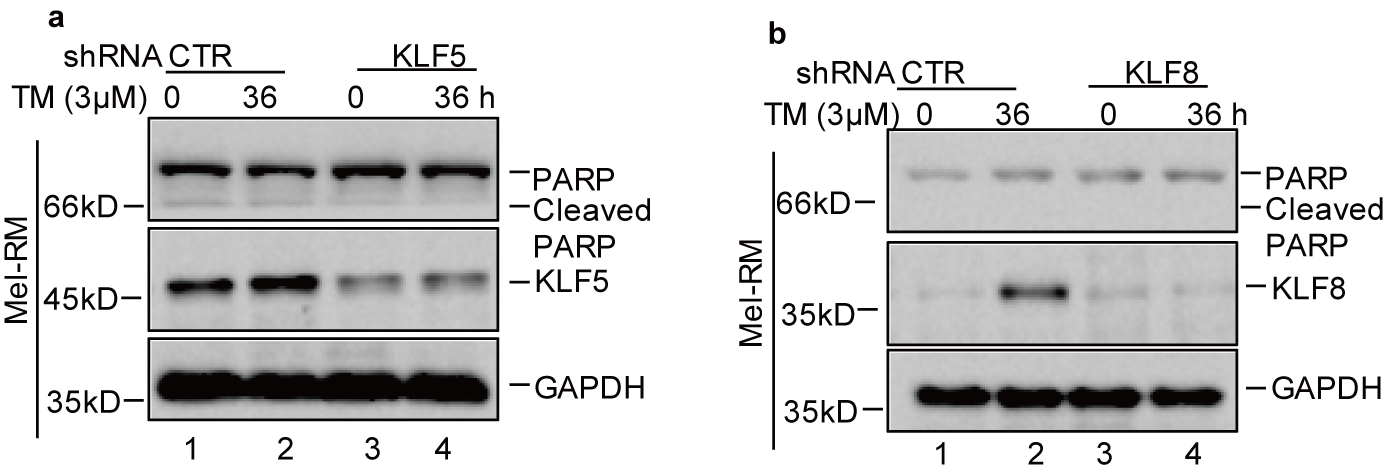

Supplement: Supplementary file 5 — Figure S3. (a-b) Mel-RM with or without KLF5 or KLF8 knockdown were treated with 3 μM TM. Cell lysates were then subjected to western blot analysis using the indicated antibodies. (TIF 192 kb) [file 13046_2018_842_MOESM5_ESM.tif]

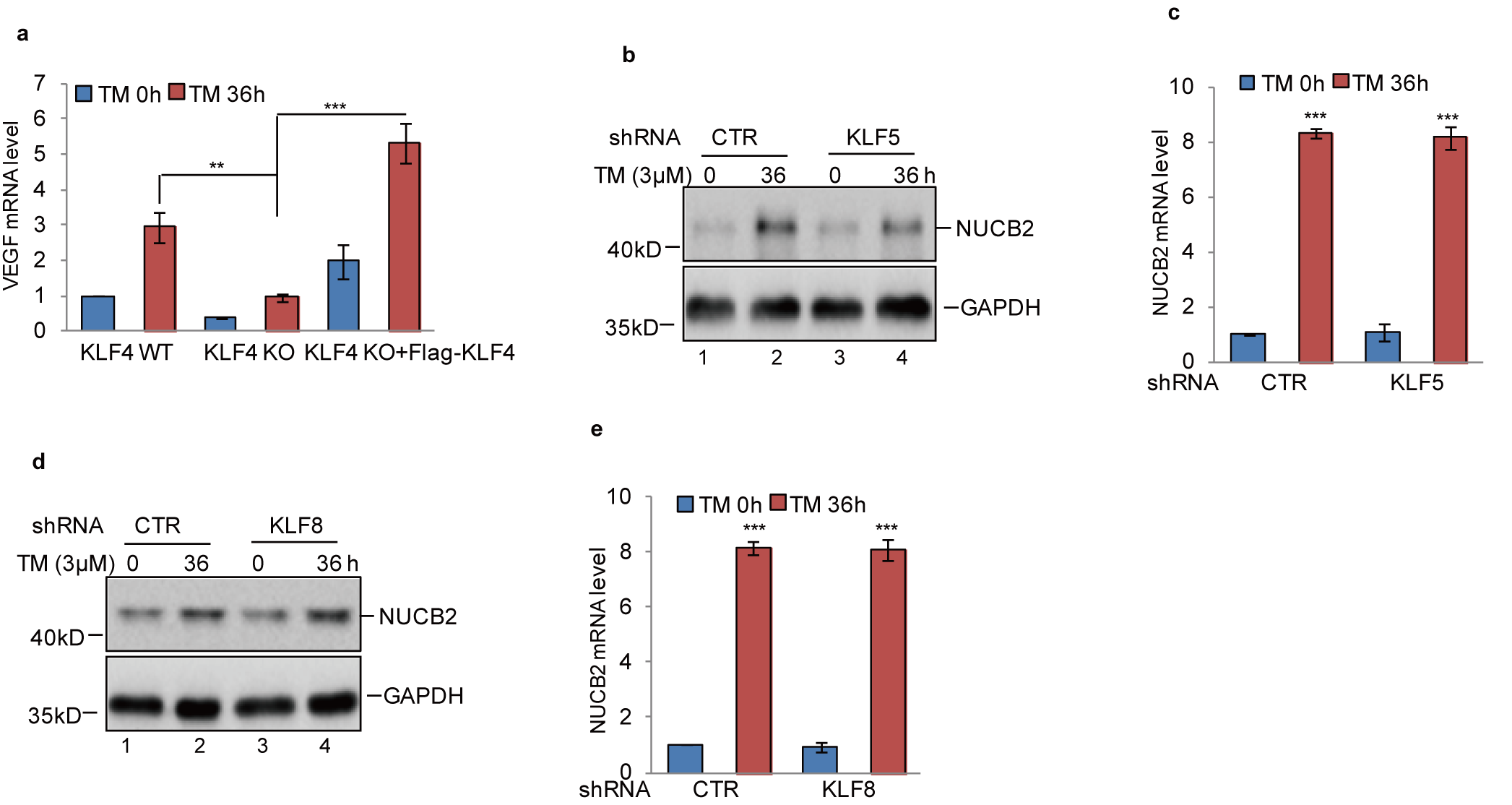

Supplement: Supplementary file 6 — Figure S4. (a) the mRNA levels of VEGF were analysed by q-RT-PCR in KLF4 WT, KLF4 KO or KLF4 KO + Flag-KLF4 Mel-RM cells with or without 3 μM TM treatment for the indicated times. The data represent the means ± SD of three independent experiments. **p < 0.01, ***p < 0.001 vs. control. (b-e) The expression levels of NUCB2 were detected by western blot and q-RT-PCR assays in Mel-RM cells with or without KLF5 or KLF8 knockdown under TM treatment. The data represent the means ± SD of three independent experiments. ***p < 0.001 vs. control. (TIF 252 kb) [file 13046_2018_842_MOESM6_ESM.tif]

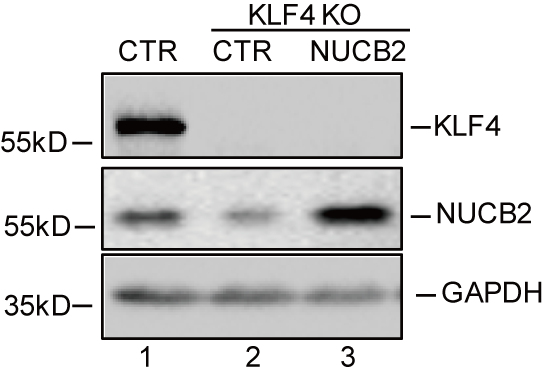

Supplement: Supplementary file 7 — Figure S5. NUCB2 was transfected in a stable manner into Mel-RM cells with or without KLF4 knockout. Cell lysates were then subjected to western blot analysis using the indicated antibodies. (TIF 69 kb) [file 13046_2018_842_MOESM7_ESM.tif]
